# Gnathostomiasis acquired after consumption of raw freshwater fish in the Amazon region: a report of two cases in Brazil

**DOI:** 10.1590/0037-8682-0127-2020

**Published:** 2020-11-13

**Authors:** Vidal Haddad, Ísis Fiorello de Oliveira, Natália Parenti Bicudo, Mariângela Esther Alencar Marques

**Affiliations:** 1 Universidade Estadual Paulista, Faculdade de Medicina de Botucatu, Botucatu, SP, Brasil.

**Keywords:** Gnasthotomiasis, Worm infestations, Fish, Peacock bass

## Abstract

Gnathostomiasis is a parasitic zoonosis caused by the helminth *Gnathostoma* spp., acquired through the consumption of raw or undercooked contaminated aquatic animals.The disease is endemic in Southeast Asia and Central America. Two male patients, both middle-aged, presented with single itchy erythemato-edematous plaques on the anterior thorax and left flank. Both had consumed raw fish in the Amazon region. The clinical and epidemiological examinations suggested gnathostomiasis, and treatment with albendazole caused total regression of the lesions. Health teams should be familiar with the disease to provide correct diagnosis. The control strategy should be based on health education for the population.

## INTRODUCTION

Gnathostomiasis is a parasitic zoonosis caused by the helminth nematode *Gnathostoma* spp., acquired by the consumption of raw or undercooked aquatic animals contaminated with larvae of *Gnasthostoma* in the third stage of development (AL3). The larvae will never become adult worms in a human host^1^ ^-^ ^6^ **.**

The disease is endemic in Southeast Asia and Central and South America. Humans are occasional hosts due to the ingestion of the larvae, which cross the digestive tract and enter the subcutaneous tissue or other solid organs (and more rarely into the central nervous system, causing the most severe forms of the disease). Cutaneous gnathostomiasis occurs three to four weeks after larvae ingestion and a migratory skin lesion ranging from a nodule to an infiltrated ill-defined mass appears. Peripheral eosinophilia and a dense eosinophilic infiltrate of the dermis and subcutaneous are suggestive of the disease, but the definitive diagnosis is made by identifying the parasite. Treatment with albendazole or ivermectin is effective, but some patients may experience recurrence even after therapy^1^ ^-^ ^6^.

Regarding the cutaneous form (the most common), a migratory skin lesion ranging from a nodule to an infiltrated ill-defined mass usually appears three to four weeks after larvae ingestion. Another presentation of the disease is the pseudo-furuncular type, which happens spontaneously or a few days after therapy and can provoke a large nodular or ill-defined infiltrated area to a tiny papule or even a pustule^7^. Peripheral eosinophilia may also occur. Histologically there is a dense eosinophilic infiltrate of the dermis and subcutaneous tissue. The definitive diagnosis is made by identifying the parasite, which is difficult. Treatment with albendazole or ivermectin is effective. Some patients may experience recurrence even after therapy^1^ ^-^ ^6^.

## CASE REPORT

**FIGURE 1:**
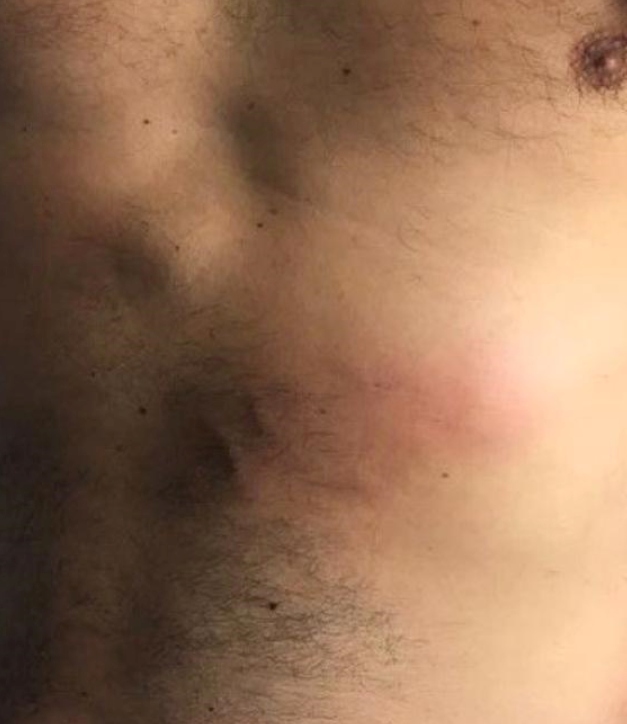
Single erythemato-edematous plaque with migratory characteristics on the anterior thorax of patient 1. Photo: Vidal Haddad Junior.

Patient 1: A middle- aged male presented with ill-defined itchy single erythemato-edematous plaques on the anterior thorax (Figure 1) with anterior diagnosis of bacterial cellulittis or paniculittis, but without fever or other systemic signs and symptoms. He reported consumption of raw *tucunarés* or peacock bass marinated in lemon juice (*ceviche*) from the Telles Pires River, on a group fishery in the Amazon region (North of the Mato Grosso State, Brazil) approximately 15 days before the exam.

**FIGURE 2:**
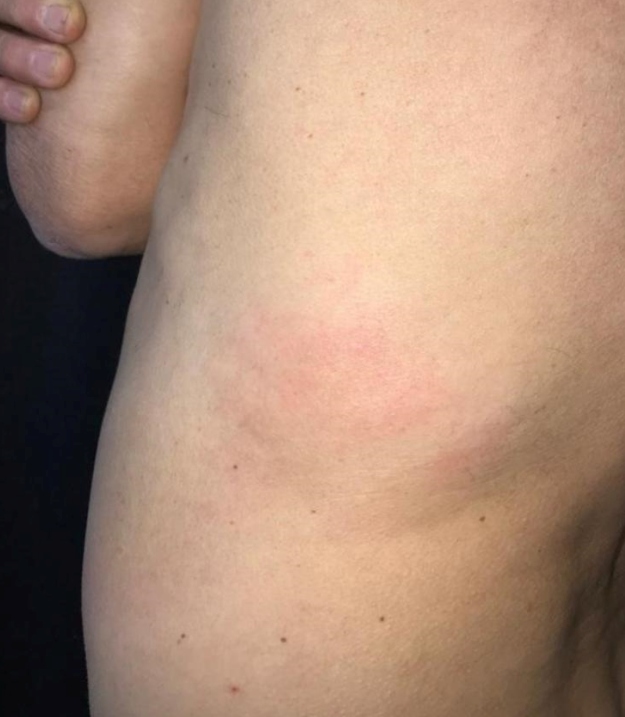
Migratory single erythemato-edematous plaque on the left flank of the patient 2. Photo: Vidal Haddad Junior.

Patient 2: A middle- aged male presented with ill-defined itchy single erythemato-edematous plaques on the left flank (Figure 2) without other systemic signs and symptoms. He also was on a group fishery and reported consumption of *tucunarés ceviche* from the Juruena River, in the same region, three weeks before the exam.

The patients were not on the same fishing expedition and were examined about two months apart.

**FIGURE 3:**
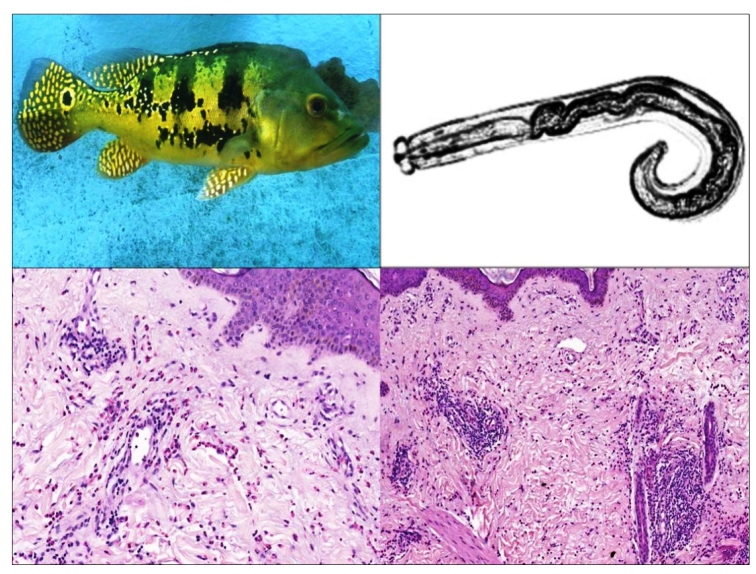
Top left: Tucunarés or peacock bass (*Cichla* sp.). Top right: schematic drawing of the *Gnasthotoma* spp. Bottom left: Histopathologic exam of patient 2 showing edema of the upper dermis and diffuse eosinophilic infiltrate (hematoxylin & eosin, ×10). Bottom right: eosinophilic infiltrate in detail (hematoxylin & eosin, ×40). Photo: Mariângela Esther Alencar Marques.

Once the clinical and epidemiological diagnoses of gnathostomiasis were made, biopsies were performed and the histopathological examinations in both patients showed a diffuse eosinophilic infiltrate reaching the dermis and subcutaneous tissue and edema of the upper dermis (Figure 3), which is very compatible with the diagnosis; however, the agent could not be visualized. The treatment with albendazole (400 mg bid) for 21 days was highly effective, and the plaques healed after two weeks.

## DISCUSSION

Both patients had consumed the meat of *tucunarés* or peacock bass (*Cichla* sp. - Figure 3) marinated in lemon juice, which is insufficient to eliminate the larvae of the parasite, unlike in meat that has been cooked, roasted, or still frozen for 3- to 5 days -20° C^3^. Although the disease is still rarely reported, there is a growing number of autochthonous cases in non-endemic countries, suggesting that the distribution of the parasite in nature may be wider than expected^1^ ^-^ ^3^ ^,^ ^5^. Health teams should be familiar with the disease for correct diagnosis and treatment. The control strategy should be based on health education for the population.
